# Exoscopic Minimally Invasive Open-Door Laminoplasty with Pedicle Screw Fixation for Cervical Ossification of the Posterior Longitudinal Ligament: A Technical Note and Preliminary Clinical Experience

**DOI:** 10.3390/jcm15062307

**Published:** 2026-03-18

**Authors:** Kentaro Yamane, Wataru Narita, Shinichiro Takao, Hisakazu Shitozawa, Kazuhiro Takeuchi, Shinnosuke Nakahara

**Affiliations:** 1 Department of Orthopaedic Surgery, National Hospital Organization Okayama Medical Center, 1711-1, Tamasu, Kita-ku, Okayama 701-1192, Okayama, Japan; shinichiro.takao1225@gmail.com (S.T.); s.hisakazu0809@gmail.com (H.S.); takeuchi.kazuhiro.qr@mail.hosp.go.jp (K.T.);; 2 Department of Orthopaedic Surgery, Kameoka Municipal Hospital, 1-1, Shinonoda, Shino-cho, Kameoka 621-8585, Kyoto, Japan; wnari77@gmail.com

**Keywords:** exoscopic minimally invasive open-door laminoplasty with pedicle screw fixation, exoscope, minimally invasive, cervical ossification of posterior longitudinal ligament, posterior decompression with instrumented fusion

## Abstract

**Background**: Posterior decompression with instrumented fusion (PDF) is a conventional surgical procedure performed in patients with massive ossification of the posterior longitudinal ligament (OPLL); however, it is invasive to the posterior cervical tissues. In this report, we introduce a novel PDF technique, exoscopic minimally invasive open-door laminoplasty with pedicle screw fixation (exLAPPS), to treat cervical OPLL, while minimizing posterior tissue damage. **Methods**: ExLAPPS was indicated for patients with K-line (−) OPLL or a canal occupying a ratio of ≥50%, allowing decompression from C3 to C7. A small midline incision was used for the navigation reference placement and exoscopic minimally invasive open-door laminoplasty, whereas bilateral lateral incisions were utilized for posterior fixation, including pedicle screw insertion, based on the minimally invasive cervical pedicle screw technique. **Results**: A total of 7 patients with K-line (-) or a canal occupancy ≥50% underwent exLAPPS for cervical OPLL. The mean operative time was 248 min (range, 165–342 min) and the mean blood loss was 320 mL (range, 50–740 mL). Postoperative imaging demonstrated adequate spinal cord decompression in all patients. A total of 52 pedicle screws were inserted, with a pedicle screw deviation rate of 1.9%. **Conclusions**: ExLAPPS is a minimally invasive surgical technique designed for posterior decompression and fixation in patients with cervical OPLL. In this preliminary case series, the procedure was successfully performed with acceptable operative time, blood loss, and screw placement accuracy. Although the present study did not include a direct comparison with conventional procedures, these preliminary observations suggest that ExLAPPS is a feasible surgical option for selected patients with cervical OPLL.

## 1. Introduction

The optimal surgical approach for cervical ossification of the posterior longitudinal ligament (OPLL) depends on both the size of the ossified mass and the cervical alignment. The K-line is a reliable indicator for determining the appropriate procedure [1]. In patients with K-line (−) OPLL, anterior cervical decompression and fusion (ADF) or posterior decompression with instrumented fusion (PDF) is commonly selected [2,3,4]. PDF is familiar to most spine surgeons and avoids anterior-approach complications such as cerebrospinal fluid leakage and dysphagia. However, it is more invasive to the posterior cervical tissues.

We previously reported a technique for reducing posterior tissue damage after cervical laminoplasty: exoscopic minimally invasive open-door laminoplasty (exLAP) [5]. The exoscope is a newly developed magnification tool featuring a compact, high-sensitivity, and high-resolution three-dimensional (3D) digital video camera and a 4K large-screen monitor [6,7]. Furthermore, minimally invasive cervical pedicle screw (MICEPS) fixation via a posterolateral approach can lessen posterior muscle injury and prevent vertebral artery damage caused by lateral pedicle screw deviation [8,9]. By integrating exLAP and MICEPS fixation, we developed a novel minimally invasive PDF technique for cervical OPLL: exoscopic minimally invasive open-door laminoplasty with pedicle screw fixation (exLAPPS). The aim of this technical report was to introduce exLAPPS and present the clinical outcomes of seven patients who underwent this procedure.

## 2. Materials and Methods

### 2.1. Surgical Indications

Similar to the indications for conventional PDF from C2–C7, patients with K-line (−) OPLL or a canal occupying ratio of ≥50% were indicated for exLAPPS. Decompression can be achieved up to C3–C7 through exLAP. Patients with vertebral artery issues that prevented pedicle screw placement or those who required screw insertion in the upper thoracic spine were excluded from this procedure.

### 2.2. Surgical Procedure

#### 2.2.1. Setup and Navigation

**Figure 1:**
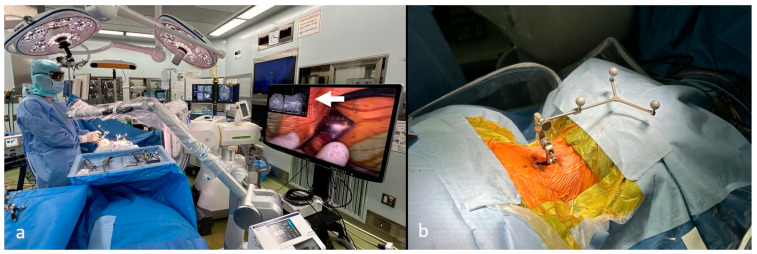
Surgical setting during exoscopic minimally invasive open-door laminoplasty (exLAP) with pedicle screw fixation. (**a**) The camera is placed above the surgical field, and the surgeon, wearing three-dimensional polarized glasses, performs the procedure while viewing the monitor. Navigation images are displayed on the exoscope monitor (white arrow). (**b**) The computed tomography navigation reference is placed through the exLAP incision.

The patient was placed in the prone position on a radiolucent carbon table using a Mayfield frame. An exoscope (ORBEYE; Sony Olympus Medical Solutions, Tokyo, Japan) was mounted above the surgical field. The surgeon wore 3D polarized glasses to operate while viewing a 4K monitor (Figure 1a). A 30–40 mm midline incision (approximately two finger-breadths) was made at C3/4. After exposing the C3–C5 spinous process, the navigation reference frame was secured (Figure 1b). Intraoperative computed tomography (CT) was then performed, and all navigated instruments were registered and verified.

#### 2.2.2. Screw and Rod Placement Using a CT-Based Navigation System

**Figure 2:**
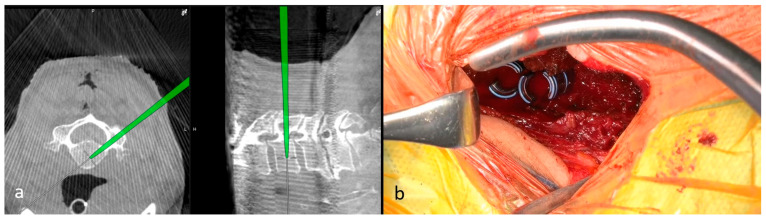
Screw placement using a computed tomography (CT)-based navigation system. (**a**) Screw insertion is performed via CT navigation. (**b**) Screws are placed through a lateral incision following the minimally invasive cervical pedicle screw fixation technique.

Two 30–50 mm lateral incisions were made for pedicle screw insertion under CT-based navigation. Following the nuchal-fascia division, the lateral mass was exposed by blunt dissection between the splenius capitis and levator scapulae. Partial detachment of the trapezius muscle was necessary when exposing the C7 lateral mass. The screw entry points were confirmed using a navigated pointer. The pedicle cortex was breached using a high-speed burr or navigated high-speed burr (Midas Rex; Medtronic, Minneapolis, MN, USA). A navigated pedicle probe or burr was then advanced along the trajectory (Figure 2a). After tapping, pedicle screws were inserted sequentially (Figure 2b) and connected with rods.

#### 2.2.3. Neural Decompression via exLAP

**Figure 3:**
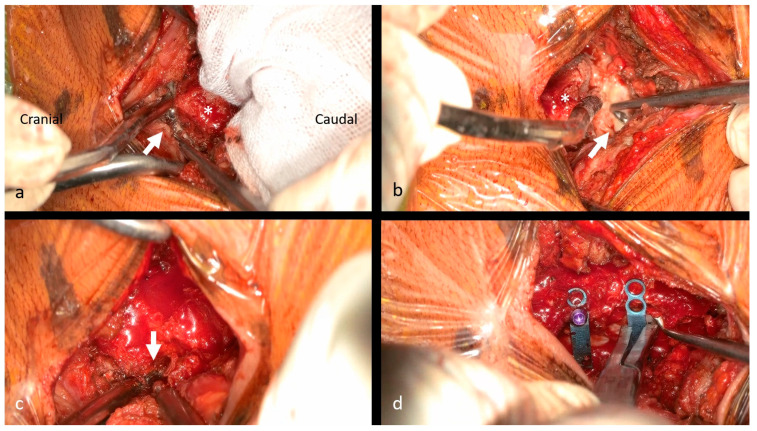
Neural decompression via exoscopic minimally invasive open-door laminoplasty. (**a**) Preserving the C2 semispinalis, the C2/3 ligamentum flavum is resected using a curved curette (white arrow). The base of the C3 spinous process (*) is visible. (**b**) The C6/7 ligamentum flavum is resected using a curved curette (white arrow). The base of the C6 spinous process (*) is visible. (**c**) A gutter is created on the open side using a high-speed burr (white arrow). (**d**) Mini-plates are placed at the elevated laminae.

Neural decompression was performed via exLAP [5]. After returning to the midline, the navigation frame was removed. The tips of the residual spinous processes were split using a high-speed burr, without dissecting the attached muscles. A deep Gelpi retractor was used to retract the paraspinal muscles. The bilateral laminae were exposed using an electric scalpel, and the spinous process bases were removed for an autograft. A fenestration was created between the C2/3 laminae with a high-speed burr, and the C2/3 ligamentum flavum was dissected using a curved curette, while completely preserving the C2 semispinalis (Figure 3a). Depending on the caudal decompression level, the flavum at C6/7 or C7/T1 was similarly released. When adequate C6/7 decompression was required while sparing the C7 spinous process and semispinalis, C7 domeplasty was performed (Figure 3b). Laminar gutters were created on both the open and hinge sides using a high-speed burr (Figure 3c). Each lamina door was elevated, and mini-plates (OPERA System; Symphony Medical Co. Ltd., Kyoto, Japan) were placed at all opened laminae (Figure 3d).

#### 2.2.4. Bone Grafting

The opened laminae and medial hinge-side lateral masses were decorticated, and a local autograft from the spinous processes was packed onto the opened laminae and medial lateral mass. After placing a drainage tube in the midline incision, all three incisions were irrigated and closed in layers. The surgical short video of ExLAPPS is available online (Supplementary Materials).

The study was approved by the Institutional Review Board of our institute, and written informed consent was obtained from all patients included in this study. The Japanese Orthopaedic Association (JOA) score for cervical myelopathy was recorded preoperatively and at one year postoperatively. The recovery rate was calculated using the formula established by Hirabayashi as follows: (postoperative JOA score − preoperative JOA score)/(17 − preoperative JOA score) × 100%. Postoperative computed tomography (CT) scans were obtained for all patients. The degree of screw deviation was classified into four grades [10]: grade 0, no deviation; grade 1, deviation < 2 mm; grade 2, deviation ≥ 2 mm and <4 mm; and grade 3, deviation ≥ 4 mm. Deviations of grade 2 or higher were defined as screw deviation. All data are expressed as the mean ± standard deviation.

## 3. Results

Seven patients with K-line (−) or a canal occupancy ≥50% underwent exLAPPS for cervical OPLL (Table 1). The mean operative time was 248 min (range, 165–342 min), and the mean blood loss was 320 mL (range, 50–740 mL). Two patients developed postoperative C5 palsy, but fully recovered with rehabilitation. In one patient, right C4 screw deviation caused C5 palsy. The deviated screw was removed during revision surgery, leading to neurological recovery with rehabilitation. The mean JOA score improved from 10.8 ± 2.4 preoperatively to 13.5 ± 2.1 at one year postoperatively, with a mean recovery rate of 45.5%. A total of 52 pedicle screws were inserted. Of these, 47 were classified as grade 0, 4 as grade 1, 1 as grade 2, and none as grade 3. Based on the definition of deviation as grade ≥ 2, the deviation rate was 1.9%.

## 4. Representative Cases

**Figure 4:**
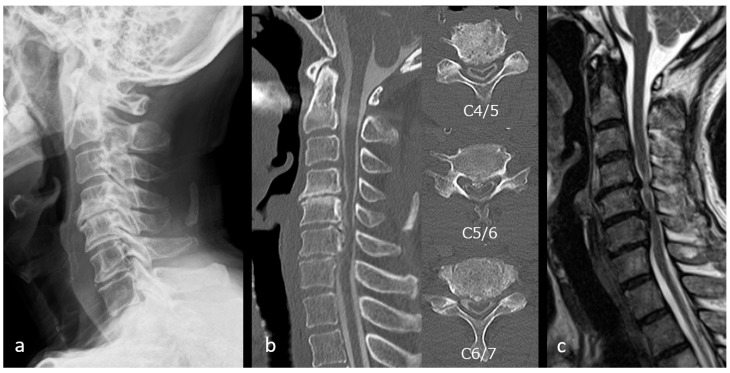
Preoperative images of Case 1. (**a**) Preoperative radiograph showing K-line (+/−). (**b**) Preoperative computed tomography images revealing ossification of the posterior longitudinal ligament at the C5/6 level with a canal occupying ratio of 58%. (**c**) Preoperative magnetic resonance image demonstrating multilevel spinal cord compression.

**Figure 5:**
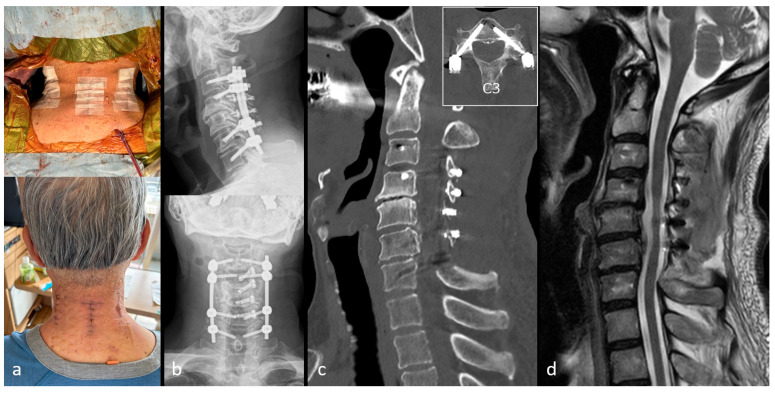
Postoperative images of Case 1. (**a**) The procedure is performed through three small incisions, each approximately two finger-breadths in length. (**b**) Postoperative radiograph. (**c**) Postoperative computed tomography images. (**d**) Postoperative magnetic resonance image demonstrating effective spinal cord decompression.

Case 1: A 70-year-old man presented with hand numbness, grip weakness, and gait disturbance. Plain radiographs showed K-line (+/−) (Figure 4a). CT revealed OPLL causing a 58% canal occupation at C5/6 (Figure 4b), and MRI demonstrated multilevel stenosis from C4/5 to C6/7 (Figure 4c). ExLAPPS was performed via a 35 mm midline and two 45 mm lateral incisions (Figure 5a). The decompression procedures included C3–C6 laminoplasty and C7 domeplasty. Posterior fusion was performed from C3 to C7 using the Cios Spin 3D C-arm (Siemens Healthineers, Forcheim, Germany) and BrainLab Kick Navigation Station (BrainLab, Munich, Germany) for data acquisition and spinal navigation. Postoperative plain radiography and CT confirmed accurate instrument placement (Figure 5b,c), and MRI revealed adequate decompression (Figure 5d). No complications were observed, and the JOA score improved from 13.5 preoperatively to 15 at one year postoperatively.

**Figure 6:**
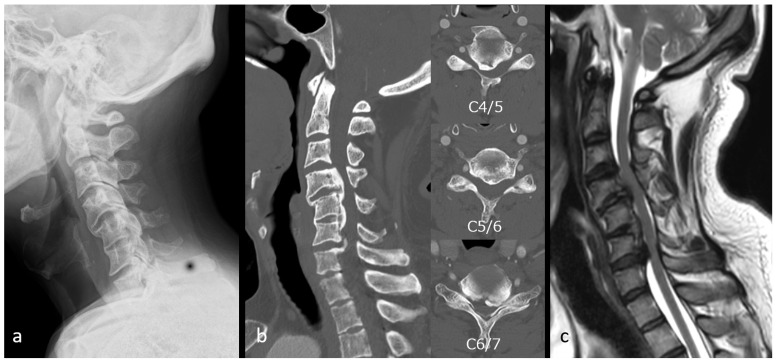
Preoperative images of Case 2. (**a**) Preoperative radiograph showing K-line (−). (**b**) Preoperative computed tomography images revealing ossification of the posterior longitudinal ligament at the C4/5 level with a canal occupying ratio of 64%. (**c**) Preoperative magnetic resonance image demonstrating multilevel spinal cord compression.

**Figure 7:**
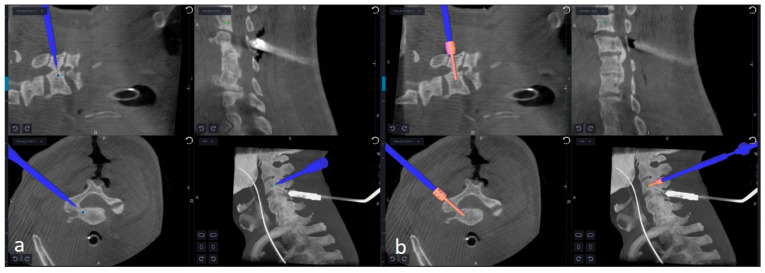
O-arm-based navigation system. (**a**) Creation of screw holes using a navigated high-speed burr. (**b**) Pedicle screw insertion via a posterolateral approach.

**Figure 8:**
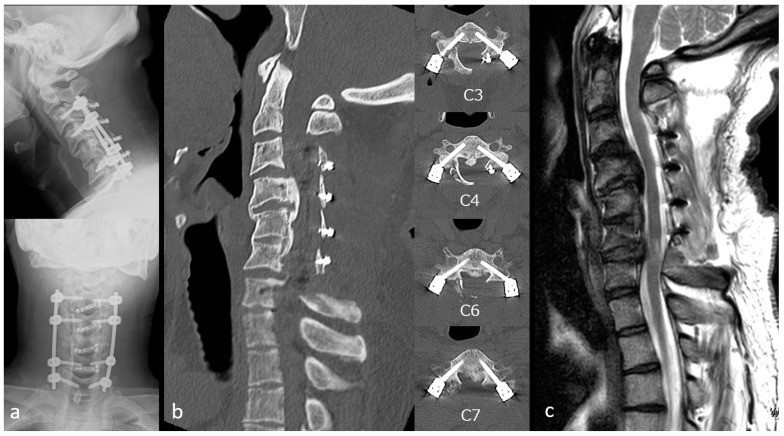
Postoperative images of Case 2. (**a**) Postoperative radiograph. (**b**) Postoperative computed tomography images. (**c**) Postoperative magnetic resonance image.

Case 2: A 44-year-old man presented with impaired fine motor function of the upper extremities and gait disturbance. Plain radiographs demonstrated a K-line (−) (Figure 6a). CT revealed OPLL with 64% canal occupation at C4/5 (Figure 6b), and MRI demonstrated multilevel stenosis from C3/4 to C6/7 (Figure 6c). ExLAPPS was performed through a 45 mm midline and two 50 mm lateral incisions. The decompression procedures consisted of C3–C6 laminoplasty and C7 domeplasty. Posterior fusion was performed from C3 to C7 using the O-arm imaging system and the StealthStation Spine 7^®^ navigation system (Medtronic, Minneapolis, MN, USA) for data acquisition and spinal navigation. The screw holes were created using a navigated high-speed burr, followed by pedicle screw insertion (Figure 7a,b). Postoperative plain radiographs and CT confirmed accurate instrument placement (Figure 8a,b), and MRI demonstrated adequate decompression (Figure 8c). No perioperative complications were observed, and the JOA score improved from 11.5 preoperatively to 15 at one year postoperatively.

## 5. Discussion

Conventional PDF for extensive cervical OPLL often requires fixation across five or more levels [3,4]. Therefore, extensive exposure of the posterior cervical tissue is required. When inserting pedicle screws, which provide stronger fixation than lateral mass screws [11], adequate cranial and caudal tissue exposure is crucial to avoid lateral screw deviation, which can lead to vertebral artery injury. Safe insertion of pedicle screws into the C3 vertebra requires exposure of the C2 lamina and, in some cases, the C1 posterior arch. However, extensive posterior soft tissue exposure increases surgical invasiveness. Therefore, there is an increasing demand for minimally invasive PDF techniques.

The surgical concept of exLAPPS focuses on minimizing posterior muscle disruption. In traditional procedures, extensive posterior soft tissue dissection is required for pedicle screw placement, which may result in severe postoperative atrophy of the posterior cervical muscles and persistent axial neck pain. Preservation of the semispinalis attachments at C2 and C7 is considered particularly important for reducing postoperative axial pain [12,13]. Since the decompression procedure in exLAPPS follows the principles of exLAP, the C2 semispinalis can be fully preserved. Furthermore, during decompression at the C6/7 intervertebral level, cranial domeplasty of the C7 lamina allows for complete preservation of the C7 semispinalis. Preserving these muscle attachments may help reduce postoperative axial pain and adjacent segment degeneration. The use of an exoscope provides a clear and safe 3D surgical view, enabling decompression from C3 to C7 through a small midline incision.

The lateral incisions used in the MICEPS fixation technique further minimize muscle injury by exploiting the intermuscular planes [8,9]. However, access to the C7 lateral mass may require a limited trapezius division. When performing intermuscular dissection, meticulous techniques are required to preserve perforating vessels and prevent massive bleeding. It is crucial to carefully dissect the muscle layers from the superficial to the deep layers. We recently transitioned from utilizing a navigated probe to a navigated high-speed burr for screw trajectory creation; unlike probing, the high-speed burr does not exert a push force on the lamina, maintaining navigation accuracy. However, the use of a navigated high-speed burr necessitates the availability of an O-arm imaging system and the StealthStation Spine 7^®^ navigation system.

The deviation rate of cervical pedicle screws using conventional methods has been reported to range widely from 2.8% to 29.1% [10,14,15]. Lateral deviations are the most common and have been attributed to factors such as vertebral body rotation and pressure from the paraspinal muscles during screw insertion [14]. The MICEPS fixation method, which utilizes a lateral skin incision, helps to avoid these issues and allows for a larger insertion angle (alpha angle), thereby reducing the risk of lateral deviation [8]. Tokioka et al. reported that the rate of grade 2 or higher deviations was 1.4% (3 of 210 screws), all of which were medial deviations [9]. In the present study, the deviation rate was 1.9%, with no cases of lateral deviation. However, one case of caudal deviation resulted in nerve root injury. In this case, the screw hole was created using a navigated pedicle probe rather than a navigated high-speed burr. In contrast, no grade 2 or higher screw deviations were observed in cases in which the screw holes were created using a navigated high-speed burr. Pedicle screw insertion in exLAPPS, similar to the MICEPS fixation method, relies heavily on navigation systems. Therefore, it is essential to recognize the limitations of navigation systems with respect to accuracy and safety.

In an effort to minimize surgical invasiveness in PDF, laminectomy combined with lateral mass screw fixation through a small midline incision may be considered an alternative approach. However, when performed through a small midline skin incision similar to that used in exLAPPS, several technical challenges may arise. First, after placement of the navigation reference frame and preparation of the screw trajectories, definitive screw placement interferes with the decompression procedure. Consequently, screws are often inserted after completion of a laminectomy. Furthermore, the navigation reference frame obstructs the operative field during decompression and needs to be removed, thereby requiring screw insertion without navigation guidance. Second, placement of rods and set screws directly over the exposed spinal cord through a limited midline incision after laminectomy may increase technical complexity and potentially elevate the risk of spinal cord injury. In contrast, in exLAPPS, instrumentation is performed through separate incisions, which may improve procedural safety. Third, bone grafting lateral to the implants through a small midline incision can be technically demanding because of the restricted working space. Considering these factors, exLAPPS may provide potential technical advantages in patients undergoing minimally invasive PDF.

Several studies have reported clinical outcomes of conventional surgical approaches for cervical OPLL, including ADF and PDF [3,4]. These procedures can achieve sufficient spinal cord decompression; however, they often require extensive posterior muscle dissection or technically demanding anterior approaches. Previous reports have suggested that posterior muscle injury may contribute to postoperative axial pain and delayed functional recovery [13]. In recent years, surgical techniques aimed at preserving posterior cervical musculature have attracted increasing attention. The ExLAPPS technique was developed based on these concepts by combining exLAP and MICEPS fixation using separate small skin incisions and intermuscular approaches. Although the present study did not include a direct comparison with conventional techniques, the surgical concept aims to minimize soft tissue disruption while allowing adequate decompression and stabilization. Future studies with larger cohorts and comparative analyses will be necessary to determine whether these theoretical advantages translate into improved clinical outcomes.

The ExLAPPS technique has a few limitations. First, it requires advanced skills in navigated pedicle screw placement following the MICEPS fixation technique and open-door laminoplasty following the exLAP method. Consequently, it is difficult to perform without navigation systems, exoscopes, or microscopes. Second, compared with conventional open surgery, performing the procedure through small skin incisions presents a learning curve for surgeons. Third, the present study includes a small number of cases, and a direct comparison of clinical outcomes with conventional methods has not been performed. Therefore, the present findings should be interpreted as preliminary observations regarding the feasibility and technical aspects of the procedure. Future studies with a larger cohort are planned to allow comparative evaluation of clinical and radiographic outcomes between exLAPPS and conventional PDF procedures. However, by overcoming these challenges, this novel surgical technique may represent a promising surgical option for the treatment of patients with cervical OPLL.

## 6. Conclusions

ExLAPPS is a novel minimally invasive PDF technique for the treatment of cervical OPLL. This approach enables complete preservation of the semispinalis attachments at C2 and C7, and pedicle screw placement is performed through intermuscular planes. In this preliminary case series, the procedure was feasible and achieved adequate decompression through limited skin incisions. This surgical concept aims to preserve the posterior cervical musculature while providing posterior stabilization. However, because this study included a limited number of cases and did not involve a direct comparison with conventional techniques, further studies are required to determine the clinical effectiveness and potential advantages of this approach.

## Figures and Tables

**Table 1:** Characteristics and surgical details of patients who underwent exoscopic minimally invasive open-door laminoplasty with pedicle screw fixation for cervical ossification of the posterior longitudinal ligament.

Patient No.	Age	Sex	K-Line	Canal Occupying Ratio (%)	Peak Level	Decompression Level	Fusion Level	Incisions (mm)		
								Midline	Right	Left
1	70	M	+/−	58	C5/6	C3–C6 + C7 domeplasty	C3–C7	35	45	45
2	73	F	+/−	55	C5/6	C4–C7	C4–C7	35	30	30
3	58	M	−	66	C4/5	C3–C7	C3–C7	40	35	35
4	63	M	+	57	C5/6	C3–C7	C3–C7	40	35	35
5	44	M	−	64	C4/5	C3–C6 + C7 domeplasty	C3–C7	45	50	50
6	76	M	+	59	C3/4	C3–C5	C3–C5	30	35	30
7	76	M	+/−	66	C4/5	C3–C6	C4–C6	35	30	30
Mean	66			61				37	37	36

## Data Availability

The data used to support the findings of this study are available from the corresponding author upon request.

## Supplementary Materials

The following supporting information can be downloaded at https://www.mdpi.com/article/10.3390/jcm15062307/s1. Surgical short video.
